# The contribution of poor and rural populations to national trends in reproductive, maternal, newborn, and child health coverage: analyses of cross-sectional surveys from 64 countries

**DOI:** 10.1016/S2214-109X(17)30077-3

**Published:** 2017-02-24

**Authors:** Cesar G Victora, Aluisio J D Barros, Giovanny V A França, Inácio C M da Silva, Liliana Carvajal-Velez, Agbessi Amouzou

**Affiliations:** a International Center for Equity in Health, Post-Graduate Program in Epidemiology, Federal University of Pelotas, Pelotas, Brazil; b Data and Analytics Unit, UNICEF, New York, NY, USA

## Abstract

**Background:**

Coverage levels for essential interventions aimed at reducing deaths of mothers and children are increasing steadily in most low-income and middle-income countries. We assessed how much poor and rural populations in these countries are benefiting from national-level progress.

**Methods:**

We analysed trends in a composite coverage indicator (CCI) based on eight reproductive, maternal, newborn, and child health interventions in 209 national surveys in 64 countries, from Jan 1, 1994, to Dec 31, 2014. Trends by wealth quintile and urban or rural residence were fitted with multilevel modelling. We used an approach akin to the calculation of population attributable risk to quantify the contribution of poor and rural populations to national trends.

**Findings:**

From 1994 to 2014, the CCI increased by 0·82 percent points a year across all countries; households in the two poorest quintiles had an increase of 0·99 percent points a year, which was faster than that for the three wealthiest quintiles (0·68 percent points). Gains among poor populations were faster in lower-middle-income and upper-middle-income countries than in low-income countries. Globally, national level increases in CCI were 17·5% faster than they would have been without the contribution of the two poorest quintiles. Coverage increased more rapidly annually in rural (0·93 percent points) than urban (0·52 percent points) areas.

**Interpretation:**

National coverage gains were accelerated by important increases among poor and rural mothers and children. Despite progress, important inequalities persist, and need to be addressed to achieve the Sustainable Development Goals.

**Funding:**

UNICEF, Wellcome Trust.

## Introduction

Since 2000, coverage levels for several reproductive, maternal, newborn, and child health interventions increased in many low-income and middle-income countries.^1^, ^2^, ^3^ However, there is growing recognition that national levels and trends could hide important inequalities that need to be tackled to achieve universal coverage.^4^, ^5^, ^6^ Whereas some countries managed to increase national-level coverage at the same time as reducing disparities among different socioeconomic groups, in other countries the magnitude of inequalities remained unchanged.^1^, ^4^

We present a comprehensive set of analyses on trends in the composite coverage index (CCI), which summarises eight interventions along the reproductive, maternal, newborn, and child health continuum of care. We focus on inequalities in terms of socioeconomic position and place of residence (urban or rural). Specifically, we estimate the proportion of the measured progress at national level that can be attributed to improvements among poor and rural inhabitants.

## Methods

### Data sources

We analysed nationally representative, cross-sectional surveys from low-income and middle-income countries. The International Center for Equity in Health's database includes 235 national surveys with reproductive, maternal, newborn, and child health indicators stratified by wealth quintile and place of residence (urban or rural). The datasets include the Demographic and Health Surveys (DHS),^7^ funded by the US Agency for International Development, and the UNICEF-supported Multiple Indicator Cluster Surveys (MICS). We used DHS data from 1994 onwards, and MICS data from 2005 onwards. These two survey programmes gather data regularly from national probability samples of households, women of reproductive age (generally aged 15–49 years), and children younger than 5 years. The random samples, generally in the thousands, are drawn with multistage cluster sampling (usually two-stage), with households drawn at the last stage. The questionnaires used in the two surveys are highly standardised.

**Research in context**

**Evidence before this study**

We searched PubMed with the search terms “intervention coverage” and “developing country” for articles published in English between Jan 1, 2005, and July 31, 2016 (the date of our final search). We identified no multicountry studies in which trends for reproductive, maternal, newborn, or child health interventions were reported according to family socioeconomic position. Studies of trends in the general population, without stratification by socioeconomic position, showed that coverage increased slowly in most countries since 2000, although some interventions showed faster gains than others. An analysis of 35 countries with two or more national surveys up to 2010 showed that countries making faster progress in coverage did so by achieving steeper increases among poor people. No investigators attempted to quantify the contribution of rural and poor families to national coverage trends.

**Added value of this study**

By pooling trends for health-intervention coverage in 64 countries from 1994 to 2014, we estimated coverage gains for different subgroups of the population, including the poorest 20%, the poorest 40%, and rural populations. We also developed methods for quantification of the contribution of these subgroups to national trends. We showed that women and children living in rural areas and those from poor families showed faster progress than the rest of the population, and thus contributed to substantial accelerations of national trends.

**Implications of all the available evidence**

Investments in reaching the poorest and rural women and children, probably driven by the incorporation of equity concerns into national programmes, seem to have paid off in terms of reducing disparities and accelerating progress at country level.

We reviewed each survey dataset rigorously to ensure that indicator numerators and denominators, and missing values, complied with the Countdown to 2015 indicator definitions. 64 low-income and middle-income countries had available data for the period Jan 1, 1994, and Dec 31, 2014, from 209 national surveys for our temporal trend analyses (appendix pp 3–4). All analyses were based on publicly available data from national surveys. Ethical clearance was the responsibility of the institutions that administered the surveys.

### Outcome variable

The CCI is a summary measure of intervention coverage along the reproductive, maternal, newborn, and child health continuum, which was developed by the Countdown to 2015 team.^2^, ^8^, ^9^ The CCI is calculated for groups of children and mothers—eg, those living in the rural area of a country, or those belonging to a specific wealth quintile. It is a weighted mean of the coverage for interventions from four domains: reproductive services (family planning coverage), maternal and newborn care (antenatal care and skilled birth attendant), immunisation (BCG; three doses of diphtheria, pertussis, and tetanus [DPT3]; and measles vaccines) and management of illness (oral rehydration therapy and care seeking for pneumonia). The four domains are equally weighted, and within each domain all indicators have the same weight, except for DPT3, which has a higher weight because three doses are needed.

The CCI is calculated by the following formula:

$$\left(\text{FPC}+\frac{\text{SBA}+\text{ANC}1}{2}+\frac{2\times \text{DPT}3+\text{BCG}+\text{MSL}}{4}+\frac{\text{ORT}+\text{CAREP}}{2}\right)$$

FPC stands for family planning coverage (also referred to as demand for family planning satisfied), SBA for skilled birth attendant, ANC1 for at least one antenatal care visit with a skilled provider, MSL for measles vaccine, ORT for oral rehydration therapy for children with diarrhoea, and CAREP for care seeking for pneumonia. Because information on care seeking for pneumonia was not collected by surveyors until the mid-1990s, the CCI time series starts in 1994.

The CCI was stratified by wealth quintiles and residence (urban *vs* rural). Wealth quintiles are derived from asset indices,^10^, ^11^ which are based on household assets (eg, radio, television, refrigerator) and characteristics of the house (eg, building materials, toilet, electricity). These variables, which are included in surveys such as the DHS and MICS, are included in a principal components analysis, a data reduction technique that produces linear combinations of the variables—so-called components, with the first component usually explaining a high proportion of data variability.^12^ This component, a continuous variable, is referred to as the wealth score. Principal component analyses are run separately for urban and rural households, and then the resulting indices are scaled so that a given score on each index means the same level of wealth.^13^ This approach is used for both the DHS and MICS.

Households are then broken into five quintiles according to the wealth score, with the lowest quintile representing the poorest 20%, and the highest quintile the richest 20%. Children are then classified into these quintiles on the basis of the wealth status of the household to which they belong. Because fertility is usually higher in the poorest households, the actual number of children for analyses tends to be higher in the poorer than in the richer quintiles. In DHS datasets, typically about 25% of the children belong to the lowest quintile and 15% to the highest (appendix p 5).

Classification of residences as urban or rural is based on boundaries defined by national authorities in each country.

### Statistical analyses

Analyses were done at the global level and by country income groups according to the World Bank 2015 classification. Aggregation at global and country income group level was done by pooling all countries with available data in each category. In the main analyses, we used unweighted means—ie, each country had the same weight.

For each subgroup of countries, we initially calculated time trends in the CCI for the whole domain. We then compared trends among the lowest quintile with those among the other four quintiles. We also compared trends in the poorest two quintiles with those in the wealthiest three quintiles, and urban trends with rural trends. We used linear multilevel regression models for each subgroup, with surveys as level one units, and countries as level two units. For country-level analyses, we used variance weighted linear regression to maximise power and precision. Models were tested for non-linearity, and in all cases the linear fit was appropriate. SEs and 95% CIs for the regression lines were calculated from the SE of the prediction.

We used the coefficients from these regressions to estimate how much the poorest quintile contributed to the overall trends in intervention coverage with an approach akin to the calculation of population attributable risk. If β_all_ is the slope of the regression (average absolute annual change) including all quintiles, and β_Q2–Q5_ is the slope of the regression excluding the poorest quintile, our measure of how much the poorest quintile contributed to the trend was defined by (β_all_ – β_Q2–Q5_)/β_all_. We used an analogous procedure to estimate the contribution of trends in the poorest 40%, and in rural areas, to the national trends.

For each survey, we calculated two wealth-based inequality indices: the slope index of inequality and the concentration index. These two indices, unlike ratio or difference between poorest and richest quintiles, account for the entire distribution of the sample by wealth score. The slope index of inequality is computed from a logistic regression model^14^ and expresses (in percent points) the absolute difference in coverage between the two extremes of the wealth distribution. The concentration index measures how far the distribution of the coverage indicator is from a totally equal distribution. It is expressed on a scale from –1 to +1, on which 0 represents equal distribution of coverage across the wealth scale. Positive concentration index values represent a pro-rich distribution, which is usually noted for health coverage indicators. The slope index of inequality expresses absolute inequality, whereas the concentration index expresses relative inequality.^14^

We also did sensitivity analyses by weighting the estimates based on national populations of children younger than 5 years in 2006,^15^ which is the median year for all surveys included in the trend analyses. The same analytic approach was used for the following individual coverage indicators: antenatal care 1+ (one or more visits to a skilled provider), antenatal care 4+ (four our more visits to any provider), skilled attendant at birth, child slept under an insecticide-treated bednet, DPT3 vaccine, oral rehydration therapy (increased fluids and continued feeding during diarrhoea), and care seeking for symptoms of pneumonia. All analyses were done in Stata (version 13.1).

### Role of the funding sources

The funding sources did not have any role in the design, conduct, analysis, or writing up of the study. The corresponding author had full access to all study data and had final responsibility for the decision to submit for publication.

## Results

Breakdowns of CCI by wealth quintile and residence were available for 209 surveys (87 surveys in 26 low-income countries, 82 surveys in 27 lower-middle-income countries, and 40 surveys in 11 upper-middle-income countries). The median number of children younger than 5 years studied increased over time from 3909 (IQR 2465–5626) in the 1990s, to 4945 (3221–8416) in 2000–09, and 5087 (3576–8842) in 2010–14.

Globally, CCI increased by 0·82 percent points a year, from 54·8% in 1994, to 71·2% in 2014 (CCI estimates at national level, stratified by wealth quintiles and place of residence, are presented in the appendix, pp 6–15). This gain was largely driven by low-income countries, where CCI increased by 0·92 percent points (Table 1, Table 2). In all country income groups, increases were faster among poor people—a finding that was particularly noticeable in lower-middle-income and upper-middle-income countries, where the slope for the lowest quintile was about 50% greater than that for the other quintiles (table 1) and the slope for the lowest two quintiles was 70% greater than that for the other three quintiles (table 2). Slopes in the lowest quintile were similar to the slopes of the two lowest quintiles combined (Table 1, Table 2), thus showing that coverage increases were alike in the two poorest quintiles. By contrast, slopes in the three richest quintiles combined were considerably less steep than those in the four richest quintiles combined (Table 1, Table 2).

**Table 1:** Trends in the composite coverage index comparing the poorest quintile with the four other quintiles, by country income groups, 1994–2014

	**Average national slope (SE)**	**Average Q1 slope (SE)**	**Average Q2–Q5 slope (SE)**	**Q1 slope/Q2–Q5 slope**	**Change in national slope due to Q1**
Global	0·82 (0·06)	1·00 (0·07)	0·75 (0·06)	1·33	8·3%
Low income	0·92 (0·11)	1·04 (0·13)	0·86 (0·11)	1·21	6·2%
Lower middle income	0·71 (0·07)	0·95 (0·11)	0·63 (0·07)	1·51	11·1%
Upper middle income	0·77 (0·09)	1·02 (0·13)	0·69 (0·09)	1·48	10·5%

Income groups are based on World Bank Data. Slopes are expressed in percent points.

**Table 2:** Trends in the composite coverage index comparing the two poorest quintiles with the three other quintiles, by country income groups, 1994–2014

	**Average national slope (SE)**	**Average Q1–Q2 slope (SE)**	**Average Q3–Q5 slope (SE)**	**Q1–Q2 slope/Q3–Q5 slope**	**Change in national slope due to Q1–Q2**
Global	0·82 (0·06)	0·99 (0·07)	0·68 (0·06)	1·46	17·5%
Low income	0·92 (0·11)	1·03 (0·12)	0·82 (0·10)	1·26	11·1%
Lower middle income	0·71 (0·07)	0·92 (0·10)	0·53 (0·06)	1·74	24·8%
Upper middle income	0·77 (0·09)	0·99 (0·10)	0·58 (0·10)	1·71	25·0%

Income groups are based on World Bank Data. Slopes are expressed in percent points.

Trends in the two poorest quintiles contributed to accelerating national trends (Table 1, Table 2). For example, in lower-middle-income and upper-middle-income countries, the actual national slopes were over 10% steeper than they would have been if changes in the lowest quintile had not been faster than those in the other quintiles (table 1). When the lowest two quintiles were examined together, national slopes in lower-middle-income and upper-middle-income groups were about 25% steeper than they would have been if changes in the lowest two quintiles were not faster than those in the other quintiles (table 2).

**Figure 1:**
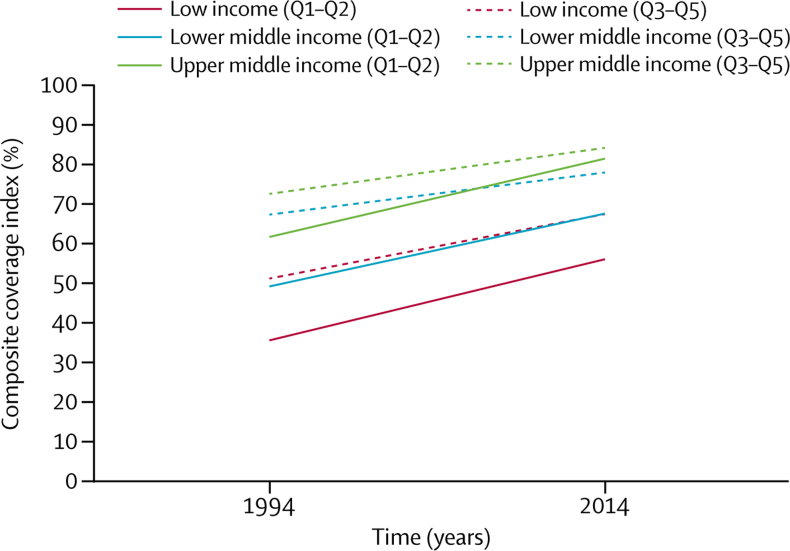
Regression lines for annual changes in composite coverage index in the two poorest quintiles and three richest quintiles of the population, by country income groups 95% CIs for the regression lines are presented in the appendix (p 22).

**Figure 2:**
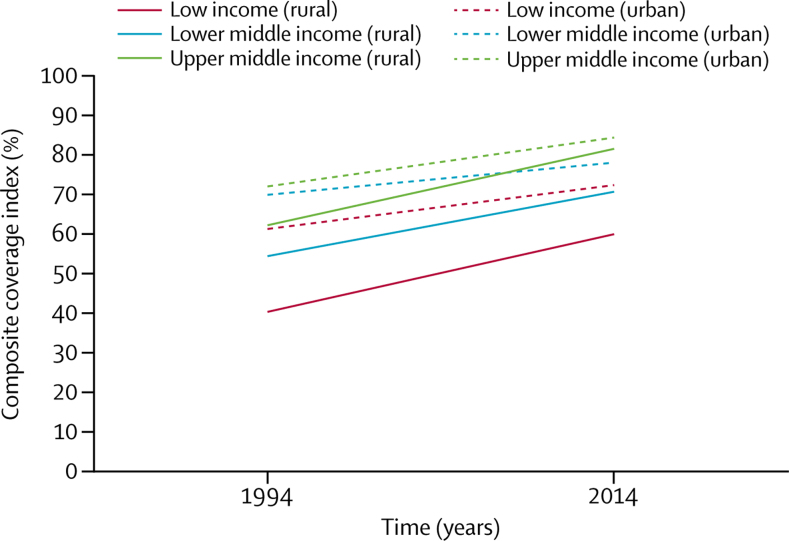
Regression lines for annual changes in composite coverage index in rural and urban areas, by country income groupings. 95% CIs for the regression lines are presented in the appendix (p 23).

Figure 1, which is based on the same regression equations as table 2, shows the initial coverage levels around 1994 and slopes over time. Inequalities tended to decrease with time, both between the three country income groupings and between the lowest two quintiles and the other quintiles within each income grouping (figure 1)—consistent with the differences in slopes (table 2). Rural areas showed faster gains in coverage than urban areas in all country groups (0·93 percent points *vs* 0·52 percent points a year), particularly in lower-middle-income countries, where the increase in coverage was 42·7% faster than it would have been in the absence of progress noted in rural areas (figure 2, table 3).

**Table 3:** Trends in urban and rural areas in the composite coverage index, by country income groups, 1994–2014

	**Average national slope (SE)**	**Average rural slope (SE)**	**Average urban slope (SE)**	**Rural slope/urban slope**	**Change in national slope due to rural areas**
Global	0·82 (0·06)	0·93 (0·07)	0·52 (0·06)	1·79	36·6%
Low income	0·92 (0·11)	0·98 (0·11)	0·55 (0·09)	1·78	39·7%
Lower middle income	0·71 (0·07)	0·82 (0·11)	0·41 (0·09)	2·00	42·7%
Upper middle income	0·77 (0·09)	0·97 (0·13)	0·62 (0·10)	1·56	20·0%

Income groups are based on World Bank Data. Slopes are expressed in percent points.

**Figure 3:**
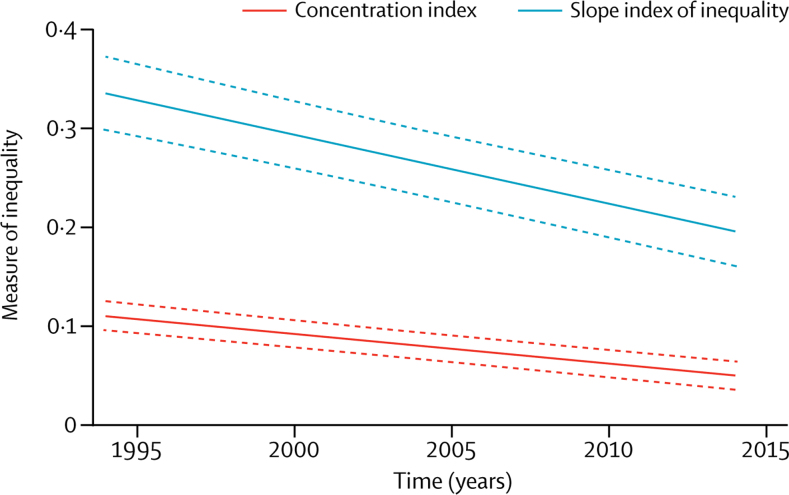
Trends in the slope index of inequality and concentration index for all countries studied, 1994–2014 Dashed lines show 95% CIs.

The narrowing of the gap between rich and poor was confirmed by analysis of the slope index of inequality and concentration index (figure 3). Both absolute (slope index of inequality) and relative (concentration index) measures of inequality for CCI substantially fell with time.

The appendix (pp 16–17, 24–26) shows results for all analyses weighted according to the national populations of children younger than 5 years in 2006. Results of weighted analyses were similar to those of unweighted analyses. In upper-middle-income countries, the contribution of poor people was more substantial in the weighted analyses, and for all countries the reduction in the indices of inequality was also faster after weighting (appendix). Regression results for all countries are shown in the appendix (pp 18–20). In most countries, slopes tended to be steeper for poor people than for rich people, and for rural populations than for urban populations, but these results should be interpreted with caution in view of the large SEs for regression slopes in some countries.

The eight interventions studied show correlation coefficients higher than 0·72 with the CCI, except for oral rehydration therapy, for which the coefficient was 0·52 (appendix p 21). Analyses based on selected individual coverage indicators including antenatal care, skilled attendance at birth, and immunisations were also done (data not shown) and provided similar results to those based on the CCI (results available upon request).

## Discussion

The CCI, as a summary measure of coverage, provides a comprehensive view of trends in inequalities and yields results that tend to be more stable than those of stand-alone coverage indicators. The mean CCI increased with time in the 64 countries included in our analyses. The increase was faster in low-income countries than in lower-middle-income and upper-middle-income countries. In most countries, progress was more substantial in the two poorest quintiles than in the rest of the population. Rates of increase in the poorest and second poorest quintiles tended to be very similar, and both rates were substantially higher than those for the three wealthier quintiles.

The coverage gap between rich and poor populations was reduced more substantially in middle-income than in low-income countries—probably because of the higher baseline coverage among wealthy people in middle-income countries, and therefore the limited room for further improvement as coverage among rich populations gets close to 100%. Nevertheless, that the average CCI level is around 80% in the wealthiest quintile in upper-middle-income countries is noteworthy—even this group has considerable room for improvement.

National coverage trends were accelerated because of the contribution of trends in the two poorest quintiles. In middle-income countries, for example, the estimated increase in national trends was around 25% faster as a result of the contribution of poor populations. Increases in coverage in rural areas were consistently faster than those in urban areas, because rural areas tend to have more poor people. Progress in rural areas had an important role in acceleration of national-level progress. These patterns were noted in most countries (appendix pp 18–20), although variability among countries was substantial.

Our analyses have several limitations. Data for trends in coverage were not available for all countries. Our analyses covered 26 of 31 low-income countries, 27 of 52 lower-middle-income countries, and 11 of 56 upper-middle-income countries, and global results should be interpreted with this limitation in mind. But our analyses of 64 countries is the largest set published so far. Our analyses covered most countries in sub-Saharan Africa and south Asia, but less than 20% of all countries in the East Asia and Pacific region and in the North Africa and Middle East region (as defined by UNICEF). Another limitation is that the CCI includes only eight interventions of the dozens that are promoted globally. However, the interventions included were those for which trend information is available for the past 20 years. Newly introduced interventions, such as postnatal care, could not be included. Reassuringly, the CCI correlates very highly in cross-sectional analyses with more complex summary coverage indicators that include many more interventions.^9^ The high correlations for nearly all eight interventions in our study shows that CCI is not being driven by only a few components.

Although two different types of survey were used (DHS and MICS), datasets were revised to ensure that indicators were uniformly compliant with international definitions. The CCI is restricted to coverage indicators that were standardised in the 1990s and for which definitions remained stable with time. The only change was for care seeking for pneumonia: in 2005, the denominator was changed to exclude children with difficulty breathing due to a blocked nose. For surveys in which both the old and new definitions could be calculated, care seeking is about 5 percent points higher with the new definition. This change, however, does not affect comparisons per wealth quintile or residence, because all groups were equally affected. Finally, asset indices have limitations but remain the method of choice for assessment of socioeconomic position from survey data in low-income and middle-income countries.^16^ The calculation of the indices takes into account the differences between assets in urban and rural areas.

During the era of the Millennium Development Goals, coverage with key reproductive, maternal, newborn, and child health interventions has increased slowly but steadily at global level.^1^, ^2^, ^3^ A detailed discussion of which factors drive coverage increases among poor populations is beyond the scope of our analyses. Country-level case studies suggest that improvements in equity result from a combination of changes in social determinants of health, pro-poor approaches in programmes in the health and other sectors, and specific targeting of health services to the geographical areas most in need.^17^, ^18^ At a global level, studies of inequalities in health are much more common than ever before,^19^ and availability of information about asset indices through national surveys has resulted in many analyses and publications, which have prompted action at many different fronts, as exemplified by the UNICEF equity-focus approach,^5^ the Every Woman Every Child Global Strategy,^20^ and the efforts led by the US Agency for International Development to incorporate equity considerations into programming.^21^

Our results are directly relevant to the achievement of several of the Sustainable Development Goals.^22^ Goal 17.18 demands analyses of progress that are disaggregated according to wealth and place of residence. Goal 3.8 promotes universal health coverage, for which our composite coverage index is a proxy. High and equitable coverage with the indicators described in this Article will contribute to progress towards goals 3.1 on maternal mortality, 3.2 on newborn and child survival, and 3.7 on sexual and reproductive health. Because of reduced infectious disease morbidity, higher intervention coverage will also contribute to the achievement of goal 2.2 on child undernutrition.

We show that national coverage gains have been driven mostly by increases among the poorest 40% of the population in low-income and middle-income countries, and by those living in rural areas. These increases are probably due to greater attention to within-country inequalities in coverage, particularly in middle-income countries. Important inequalities persist, and need to be addressed to reduce child deaths further.

For the **International Center for Equity in Health's database** see http://equidade.org

For the **Multiple Indicator Cluster Surveys** see http://mics.unicef.org/

For the **Countdown to 2015 indicator definitions** see http://www.countdown2015mnch.org

For the **World Bank 2015 classification** see http://databank.worldbank.org
