# Supplementary material for: The contribution of poor and rural populations to national trends in reproductive, maternal, newborn, and child health coverage: analyses of cross-sectional surveys from 64 countries
Source: Lancet Glob Health. 2017 Feb 24;5(4):e402–7. doi: 10.1016/S2214-109X(17)30077-3 (PMC5565524; doi:10.1016/S2214-109X(17)30077-3)

THE LANCET
Global Health

Supplementary appendix

This appendix formed part of the original submission and has been peer reviewed. We post it as supplied by the authors.

Supplement to: Victora CG, Barros AJD, França GVA, da Silva ICM, Carvajal-Velez L, Amouzou A. The contribution of poor and rural populations to national trends in reproductive, maternal, newborn, and child health coverage: analyses of cross-sectional surveys from 64 countries. *Lancet Glob Health* 2017; published online Feb 23. http://dx.doi.org/10.1016/S2214-109X(17)30077-3.

THE LANCET Global Health

Supplementary appendix

This appendix formed part of the original submission and has been peer reviewed. We post it as supplied by the authors.

Supplement to: Victora CG, et al. The contribution of poor and rural populations to national trends in reproductive, maternal, newborn and child health coverage: analyses of cross-sectional surveys from 64 countries. Lancet Glob Health 2016.

Index to the supplementary appendix

Tables

Table S1. List of countries and surveys included in the analyses. 3

Table S2. Distribution of the children studied in 64 countries, by quintiles of household wealth.........5

Table S3. Composite coverage index at national level and stratified by wealth quintiles and place of residence for each country and year of survey. 6

Table S4. Time trends in the composite coverage index comparing (a) the poorest quintile (Q1) with the four richest quintiles (Q2-Q5), and (b) the two poorest (Q1-Q2) with the three wealthiest quintiles (Q3-Q4), by country income groups, 1994-2014. Slopes are expressed in percent points. Analyses weighted by national under-five populations. 16

Table S5. Trends in urban and rural areas in the composite coverage index, by country income groups, 1994-2014. Slopes are expressed in percent points. Analyses weighted by national under-five populations. 17

Table S6. Trends in the composite coverage index in the 40% poorest (Q1-Q2), the 60% richest (Q3-Q5), urban and rural areas, by country, 1994-2014. Slopes are expressed in percent points. 18

Table S7. Pearson correlation matrix for CCI and the eight separate indicators included in this index, in the most recent survey in the 64 countries under study. 21

Figures

Figure S1. Regression lines for annual changes in CCI in the poorest 40% (Q1-Q2) and richest 60% (Q3-Q5) of the population, by country income groups, showing confidence intervals. 22

Figure S2. Regression lines for annual changes in CCI in rural and urban areas, by country income groups, showing confidence intervals...23

Figure S3. Regression lines for annual changes in CCI according to wealth quintiles, by country income groupings (low, lower middle and upper middle income). Analyses weighted by national under-five populations. Comparison in the poorest 40% (Q1-Q2) and richest 60% (Q3-Q5). 24

Figure S4. Regression lines for annual changes in CCI according in rural and urban areas, by country income groupings. Analyses weighted by national under-five populations.......................................25

Figure S5. Time trends in CCI and CIX for all countries studied, 1994-2014. Analyses weighted by national under-five populations...26

Tables

Table S1. List of countries and surveys included in the analyses.

Country	Earliest survey	2nd survey	3rd survey	4th survey	5th survey
**Armenia**	2000	2005	2010		
**Bangladesh**	1999	2004	2007	2011	
**Belize**	2006	2011			
**Benin**	1996	2001	2006	2011	
**Bolivia**	1994	1998	2003	2008	
**Bosnia and Herzegovina**	2006	2011			
**Burkina Faso**	2003	2006	2010		
**Cambodia**	2000	2005	2010	2014	
**Cameroon**	1998	2004	2006	2011	
**CAR**	1994	2006	2010		
**Chad**	1996	2004	2010		
**Colombia**	1995	2000	2005	2010	
**Comoros**	1996	2012			
**Congo Brazzaville**	2005	2011			
**Congo Democratic Republic**	2007	2010	2013		
**Cote d'Ivoire**	1994	1998	2006	2011	
**Dominican Republic**	1996	1999	2002	2007	2013
**Egypt**	1995	2000	2005	2008	2014
**Ethiopia**	2000	2005	2011		
**Gabon**	2000	2012			
**Ghana**	1998	2003	2008	2011	2014
**Guatemala**	1995	1998			
**Guinea**	1999	2005	2012		
**Guyana**	2006	2009			
**Haiti**	2000	2005	2012		
**Honduras**	2005	2011			
**India**	1998	2005			
**Indonesia**	1997	2002	2007	2012	
**Jordan**	1997	2002	2007	2012	
**Kazakhstan**	1995	1999	2010		
**Kenya**	1998	2003	2008		
**Kyrgyzstan**	1997	2005	2012	2014	
**Lesotho**	2004	2009			
**Liberia**	2007	2013			
**Macedonia**	2005	2011			
**Madagascar**	1997	2003	2008		
**Malawi**	2000	2004	2010	2013	
**Mali**	1995	2001	2006	2012	
**Mauritania**	2007	2011			

Table S2. List of countries and surveys included in the analyses. (continued)

Country	Earliest survey	2nd survey	3rd survey	4th survey	5th survey
**Moldova**	2005	2012			
**Mongolia**	2005	2010			
**Montenegro**	2005	2013			
**Mozambique**	1997	2003	2011		
**Namibia**	2000	2006	2013		
**Nepal**	1996	2001	2006	2011	2014
**Nicaragua**	1998	2001			
**Niger**	1998	2006	2012		
**Nigeria**	2003	2007	2008	2011	2013
**Pakistan**	2006	2012			
**Peru**	1996	2000	2004/2012*		
**Philippines**	1998	2003	2008	2013	
**Rwanda**	2000	2005	2010		
**Senegal**	2005	2010	2012	2014	
**Sierra Leone**	2008	2010	2013		
**Swaziland**	2006	2010			
**Tajikistan**	2005	2012			
**Tanzania**	1996	1999	2004	2010	
**Togo**	1998	2006	2010	2013	
**Uganda**	1995	2000	2006	2011	
**Uzbekistan**	1996	2006			
**Vietnam**	1997	2002	2010	2013	
**Yemen**	2006	2013			
**Zambia**	1996	2001	2007	2013	
**Zimbabwe**	1994	1999	2005	2010	2014

* Continuous DHS based on yearly data collection

Table S2. Distribution of the children studied in 64 countries, by quintiles of household wealth.

Quintile	Mean proportion (%)	Range (%)	
		Low	High
**Q1 (poorest)**	24.5	12.3	35.9
**Q2**	21.9	11.4	27.5
**Q3**	20.2	16.2	27.0
**Q4**	18.6	13.1	24.0
**Q5 (wealthiest)**	14.7	8.6	30.2

Table S3. Composite coverage index at national level and stratified by wealth quintiles and place of residence for each country and year of survey.

Country	Year	Composite coverage Index (%)							
		National	Wealth quintiles					Place of residence	
			Q1 (poorest)	Q2	Q3	Q4	Q5 (richest)	Rural	Urban
**Low income countries**									
**Bangladesh**	1999	52.6	43.1	48.4	51.7	55.2	71.4	49.9	65.7
**Bangladesh**	2004	59.1	48.6	55.7	58.7	63.9	76.4	56.8	68.7
**Bangladesh**	2007	66.8	59.3	62.2	66.8	70.0	79.1	64.8	73.9
**Bangladesh**	2011	68.4	60.6	64.3	66.8	72.5	81.2	65.8	77.8
**Benin**	1996	52.3	37.7	46.9	55.1	60.0	69.1	49.7	58.3
**Benin**	2001	59.0	49.2	51.9	60.8	64.5	71.4	56.9	63.4
**Benin**	2006	57.4	44.2	52.1	56.9	62.0	72.2	53.8	63.5
**Benin**	2011	57.6	45.3	54.1	60.1	61.9	66.2	55.3	61.0
**Burkina Faso**	2003	50.4	39.3	43.0	47.9	51.4	73.7	46.3	73.3
**Burkina Faso**	2006	56.4	51.4	50.2	52.3	56.3	70.5	52.2	69.3
**Burkina Faso**	2010	64.6	52.1	57.0	63.4	67.9	78.3	60.7	76.8
**CAR**	1994	48.9	34.9	41.2	47.8	56.5	65.8	40.0	61.6
**CAR**	2006	50.2	34.1	40.2	48.1	59.2	68.9	40.7	63.4
**CAR**	2010	44.7	32.4	34.5	40.1	54.2	65.7	35.2	60.0
**Cambodia**	2000	46.0	34.0	38.9	44.6	50.8	71.4	44.1	57.5
**Cambodia**	2005	62.7	52.5	57.8	63.7	69.7	75.7	61.6	68.8
**Cambodia**	2010	75.3	67.4	71.6	79.5	79.1	81.9	74.1	81.7
**Cambodia**	2014	80.4	74.0	78.2	81.9	83.4	85.9	79.6	84.9
**Chad**	1996	26.2	15.5	18.5	19.4	28.7	47.9	20.0	46.4
**Chad**	2004	28.5	15.6	22.0	26.7	30.8	46.1	24.2	46.2
**Comoros**	1996	53.7	41.0	50.7	54.3	57.8	71.1	51.8	59.6

Table S3. Composite coverage index at national level and stratified by wealth quintiles and place of residence for each country and year of survey. (continued)

Country	Year	Composite coverage Index (%)							
		National	Wealth quintiles					Place of residence	
			Q1 (poorest)	Q2	Q3	Q4	Q5 (richest)	Rural	Urban
**Comoros**	2012	62.1	49.5	58.5	64.6	71.8	68.9	58.1	70.7
**Congo Democratic Republic**	2007	56.2	47.0	50.4	53.9	60.5	71.9	50.9	64.0
**Congo Democratic Republic**	2010	57.0	51.3	53.3	53.4	60.1	68.9	54.1	64.7
**Congo Democratic Republic**	2013	59.2	49.3	55.3	58.5	63.1	71.2	55.2	66.9
**Ethiopia**	2000	22.5	15.4	15.1	17.7	20.8	45.2	18.2	54.0
**Ethiopia**	2005	26.6	16.8	18.3	25.4	26.4	47.8	23.1	59.3
**Ethiopia**	2011	37.4	24.4	31.0	32.2	39.5	65.1	32.7	63.0
**Guinea**	1999	41.3	26.8	30.3	38.6	50.9	63.5	34.2	60.0
**Guinea**	2005	47.1	35.3	40.1	47.3	55.1	63.8	42.6	60.9
**Guinea**	2012	46.1	31.4	42.9	44.9	52.8	64.6	40.6	61.4
**Haiti**	2000	44.8	33.7	38.8	45.5	49.7	61.4	40.3	54.3
**Haiti**	2005	48.9	37.8	42.3	50.5	55.0	63.7	44.9	56.6
**Haiti**	2012	57.5	49.4	51.3	59.6	62.9	67.0	54.3	62.8
**Kenya**	1998	66.3	53.4	61.6	66.3	71.7	81.1	63.6	77.6
**Kenya**	2003	61.2	45.3	55.9	63.6	69.4	73.7	58.7	71.5
**Kenya**	2008	67.4	55.4	61.7	69.7	71.5	76.9	65.3	76.0
**Liberia**	2007	51.0	37.5	39.0	54.1	61.2	70.3	44.0	65.5
**Liberia**	2013	61.4	52.0	59.7	62.4	67.2	68.8	56.9	65.6
**Madagascar**	1997	46.3	31.1	35.7	40.2	55.6	73.4	41.8	60.1
**Madagascar**	2003	57.3	40.6	46.5	54.2	67.5	81.2	53.3	73.1
**Madagascar**	2008	63.2	47.3	54.0	64.3	72.9	82.8	60.9	78.6
**Malawi**	2000	62.2	55.3	59.8	59.2	64.9	75.2	60.5	74.3

Table S3. Composite coverage index at national level and stratified by wealth quintiles and place of residence for each country and year of survey. (continued)

Country	Year	Composite coverage Index (%)							
		National	Wealth quintiles					Place of residence	
			Q1 (poorest)	Q2	Q3	Q4	Q5 (richest)	Rural	Urban
**Malawi**	2004	64.6	56.5	60.6	63.3	69.3	75.9	63.0	75.6
**Malawi**	2010	75.2	70.3	73.3	75.0	77.1	81.0	74.6	78.4
**Malawi**	2013	80.0	76.7	79.3	81.1	80.8	83.0	79.8	81.2
**Mali**	1995	34.9	17.9	25.0	29.8	39.4	62.5	26.3	57.4
**Mali**	2001	38.5	27.9	29.0	29.1	41.3	66.5	30.5	62.1
**Mali**	2006	44.7	35.9	38.1	39.4	45.9	64.3	38.7	58.9
**Mali**	2012	49.4	34.6	42.1	44.0	58.3	66.9	44.7	66.2
**Mozambique**	1997	44.4	24.4	36.7	40.6	56.2	67.2	36.4	68.5
**Mozambique**	2003	63.3	50.5	57.9	63.1	70.5	80.4	58.0	75.3
**Mozambique**	2011	60.2	47.0	51.1	58.8	67.1	76.0	54.5	72.3
**Nepal**	1996	35.2	24.4	30.4	33.5	38.1	55.6	33.5	57.0
**Nepal**	2001	49.8	39.5	43.7	48.2	54.9	69.4	48.2	69.1
**Nepal**	2006	56.8	42.6	52.0	57.0	63.4	75.0	54.7	70.7
**Nepal**	2011	63.6	52.4	58.8	62.6	71.9	81.3	61.9	78.9
**Nepal**	2014	68.4	58.9	65.6	66.2	74.1	82.4	66.5	80.9
**Niger**	1998	33.6	21.3	24.6	25.6	34.2	63.0	26.3	65.7
**Niger**	2006	40.6	33.4	35.0	33.3	39.4	62.3	36.0	63.8
**Niger**	2012	56.3	45.0	51.1	52.6	58.0	72.7	52.7	75.2
**Rwanda**	2000	48.9	43.7	45.4	48.2	48.8	61.1	46.7	60.8
**Rwanda**	2005	53.4	48.0	50.6	51.7	53.4	64.3	51.8	62.7
**Rwanda**	2010	72.9	68.3	70.5	73.1	74.2	79.9	71.9	78.2
**Sierra Leone**	2008	51.2	43.6	46.9	49.1	53.7	64.0	47.1	61.2

Table S3. Composite coverage index at national level and stratified by wealth quintiles and place of residence for each country and year of survey. (continued)

Country	Year	Composite coverage Index (%)							
		National	Wealth quintiles					Place of residence	
			Q1 (poorest)	Q2	Q3	Q4	Q5 (richest)	Rural	Urban
**Sierra Leone**	2010	62.7	57.3	58.2	61.6	66.5	70.0	60.6	67.2
**Sierra Leone**	2013	66.8	63.9	63.9	64.7	69.4	73.0	64.6	72.2
**Tajikistan**	2005	68.1	61.0	65.0	67.0	70.5	75.9	66.7	72.2
**Tajikistan**	2012	74.0	72.3	71.7	77.2	75.1	75.2	73.2	76.3
**Tanzania**	1996	59.9	48.4	54.9	55.5	62.7	76.8	55.9	75.5
**Tanzania**	1999	60.5	48.7	58.3	56.3	66.1	74.5	56.7	74.6
**Tanzania**	2004	66.7	56.6	62.9	64.2	69.5	81.5	63.0	79.6
**Tanzania**	2010	71.1	61.8	66.5	66.1	76.7	86.3	67.4	82.9
**Togo**	1998	46.2	35.1	41.8	47.3	51.1	63.6	41.6	61.1
**Togo**	2006	50.5	41.3	44.1	47.1	57.7	62.9	46.3	57.5
**Togo**	2010	49.6	36.9	44.2	48.6	58.8	65.1	43.6	62.3
**Togo**	2013	57.2	49.6	50.7	53.1	62.4	71.4	51.7	67.0
**Uganda**	1995	51.7	43.1	45.7	49.3	54.2	67.1	49.3	68.1
**Uganda**	2000	52.5	51.0	61.1	52.0	45.8	54.1	49.8	70.6
**Uganda**	2006	58.8	53.0	52.1	56.1	61.5	71.5	56.9	71.4
**Uganda**	2011	65.0	58.0	61.0	63.1	66.4	75.7	62.9	76.2
**Zimbabwe**	1994	73.4	66.0	69.1	72.6	78.7	81.5	70.8	80.1
**Zimbabwe**	1999	77.7	74.7	75.2	72.5	78.7	86.7	74.2	84.5
**Zimbabwe**	2005	66.9	56.5	64.3	68.4	71.5	79.6	63.3	76.0
**Zimbabwe**	2010	70.5	65.1	66.4	70.7	75.2	74.9	68.7	74.2
**Zimbabwe**	2014	80.5	75.7	78.3	80.0	83.5	85.8	79.1	84.3

Table S3. Composite coverage index at national level and stratified by wealth quintiles and place of residence for each country and year of survey. (continued)

Country	Year	Composite coverage Index (%)							
		National	Wealth quintiles					Place of residence	
			Q1 (poorest)	Q2	Q3	Q4	Q5 (richest)	Rural	Urban
**Lower-middle income countries**									
**Armenia**	2000	75.4	71.5	77.1	74.5	78.9	75.9	75.3	75.5
**Armenia**	2005	76.5	76.3	69.3	77.1	77.4	87.7	77.0	74.8
**Armenia**	2010	85.7	84.7	81.1	83.7	87.7	86.8	86.9	85.6
**Bolivia**	1994	52.2	30.8	44.5	52.4	65.2	80.0	39.6	62.3
**Bolivia**	1998	56.6	36.2	49.5	59.0	70.5	77.4	41.9	66.2
**Bolivia**	2003	67.8	53.4	64.5	68.3	76.3	84.2	59.3	73.5
**Bolivia**	2008	71.3	60.0	68.6	74.1	76.9	81.1	64.9	76.3
**Cameroon**	1998	52.0	33.3	37.5	53.1	66.6	72.1	45.1	67.5
**Cameroon**	2004	60.5	42.3	50.1	63.4	70.2	77.6	51.5	69.8
**Cameroon**	2006	70.3	57.1	69.2	72.6	77.9	84.9	64.8	78.6
**Cameroon**	2011	59.2	31.2	55.1	64.2	69.6	75.8	50.3	69.5
**Congo Brazzaville**	2005	68.4	57.0	63.8	71.0	75.6	78.7	62.0	75.1
**Congo Brazzaville**	2011	72.8	62.9	69.5	74.2	78.5	81.4	65.9	76.9
**Cote dIvoire**	1994	45.3	25.8	35.7	43.3	55.1	67.4	35.9	60.4
**Cote dIvoire**	1998	50.6	32.7	44.9	46.8	61.2	74.2	43.1	65.1
**Cote dIvoire**	2006	56.6	42.7	50.6	55.8	66.6	76.9	48.8	69.6
**Cote dIvoire**	2011	55.5	43.7	50.8	53.7	62.7	69.3	48.8	65.6
**Egypt**	1995	62.2	48.0	54.3	62.3	69.7	78.3	55.9	70.9
**Egypt**	2000	72.1	61.6	67.4	72.3	77.7	81.5	68.1	78.2
**Egypt**	2005	75.8	68.0	72.9	76.3	79.5	82.5	73.0	80.0
**Egypt**	2008	77.3	70.1	74.7	77.4	80.5	83.2	74.6	81.3

Table S3. Composite coverage index at national level and stratified by wealth quintiles and place of residence for each country and year of survey. (continued)

Country	Year	Composite coverage Index (%)							
		National	Wealth quintiles					Place of residence	
			Q1 (poorest)	Q2	Q3	Q4	Q5 (richest)	Rural	Urban
**Egypt**	2014	79.1	74.7	77.2	81.1	81.0	80.6	78.7	80.1
**Ghana**	1998	52.0	39.8	45.9	51.5	59.5	71.8	47.5	64.3
**Ghana**	2003	59.3	45.8	55.3	61.1	65.5	77.2	53.4	70.1
**Ghana**	2008	64.0	52.5	60.0	63.5	72.8	78.7	60.1	70.3
**Ghana**	2011	69.1	60.1	65.2	69.1	75.5	77.7	64.8	76.2
**Ghana**	2014	67.9	62.5	63.4	69.4	71.3	74.2	65.7	71.0
**Guatemala**	1995	50.1	31.4	37.3	48.1	60.3	77.9	42.2	62.6
**Guatemala**	1998	55.7	35.2	43.0	54.3	66.6	77.1	48.3	66.3
**Guyana**	2006	67.4	60.8	67.8	69.3	71.9	71.3	66.4	71.0
**Guyana**	2009	72.6	64.5	73.9	80.6	73.5	71.9	72.0	74.3
**Honduras**	2005	76.2	67.5	73.6	77.8	81.9	84.3	72.4	81.4
**Honduras**	2011	83.7	79.4	82.7	85.1	85.5	87.3	82.1	85.5
**India**	1998	57.7	41.0	48.6	59.4	68.3	78.3	53.4	71.9
**India**	2005	64.1	48.9	57.7	65.3	73.8	82.8	60.0	75.4
**Indonesia**	1997	70.3	58.0	65.7	71.2	76.4	84.5	66.9	79.3
**Indonesia**	2002	73.6	62.1	69.4	75.0	79.7	84.1	69.0	78.7
**Indonesia**	2007	76.4	63.9	74.6	79.1	82.6	83.6	73.3	80.9
**Indonesia**	2012	80.4	68.9	81.4	83.6	85.5	83.7	78.1	82.8
**Kyrgyzstan**	1997	75.0	70.7	78.0	76.2	73.7	82.5	73.4	82.9
**Kyrgyzstan**	2005	71.0	68.1	67.9	70.6	68.4	77.7	67.7	76.1
**Kyrgyzstan**	2012	76.4	76.2	77.4	76.5	71.8	82.6	75.5	78.1
**Kyrgyzstan**	2014	73.8	72.5	70.9	73.0	77.3	78.2	72.9	77.2

Table S3. Composite coverage index at national level and stratified by wealth quintiles and place of residence for each country and year of survey. (continued)

Country	Year	Composite coverage Index (%)							
		National	Wealth quintiles					Place of residence	
			Q1 (poorest)	Q2	Q3	Q4	Q5 (richest)	Rural	Urban
**Lesotho**	2004	67.6	55.9	60.7	68.8	72.4	79.6	65.7	77.1
**Lesotho**	2009	71.5	59.1	67.1	73.0	76.1	79.7	68.2	81.2
**Mauritania**	2007	50.7	33.8	42.5	51.5	59.7	64.6	42.6	59.7
**Mauritania**	2011	50.1	35.7	42.8	51.6	60.4	61.9	43.6	59.7
**Moldova**	2005	84.6	81.9	87.3	87.4	81.8	86.1	85.0	84.2
**Moldova**	2012	87.3	88.7	89.7	86.5	88.0	85.6	89.1	84.3
**Mongolia**	2005	82.0	79.5	80.3	84.2	84.2	83.6	81.2	82.6
**Mongolia**	2010	87.3	82.9	87.5	89.4	85.0	89.3	85.6	87.7
**Nicaragua**	1998	71.9	58.0	69.8	75.9	81.7	80.1	63.8	78.5
**Nicaragua**	2001	77.8	66.6	78.7	81.2	81.3	86.5	73.0	82.4
**Nigeria**	2003	38.2	21.2	24.2	34.3	44.8	70.5	31.5	52.5
**Nigeria**	2007	40.8	16.2	26.1	34.9	51.8	66.1	59.0	32.1
**Nigeria**	2008	41.5	17.2	26.5	41.9	56.8	72.1	33.6	58.1
**Nigeria**	2011	47.3	22.8	34.9	48.9	59.9	74.2	40.2	62.5
**Nigeria**	2013	43.3	14.6	27.0	44.3	57.8	75.5	31.5	62.6
**Pakistan**	2006	55.9	37.1	46.8	56.1	66.7	75.0	50.5	67.3
**Pakistan**	2012	62.2	43.8	55.4	64.0	71.8	79.3	57.2	73.3
**Philippines**	1998	69.0	55.1	65.3	74.8	78.9	80.1	62.9	75.9
**Philippines**	2003	73.9	59.8	71.8	79.5	81.5	86.7	68.0	80.1
**Philippines**	2008	72.3	59.0	71.5	76.1	80.8	81.8	68.0	76.9
**Philippines**	2013	76.9	67.7	76.4	80.9	82.0	82.4	74.3	79.8
**Senegal**	2005	55.7	43.5	48.0	54.5	63.9	69.7	48.7	65.3

Table S3. Composite coverage index at national level and stratified by wealth quintiles and place of residence for each country and year of survey. (continued)

Country	Year	Composite coverage Index (%)							
		National	Wealth quintiles					Place of residence	
			Q1 (poorest)	Q2	Q3	Q4	Q5 (richest)	Rural	Urban
**Senegal**	2010	60.2	46.2	54.0	62.7	64.6	74.1	53.0	68.8
**Senegal**	2012	62.2	47.4	56.0	62.7	70.3	74.7	55.1	72.5
**Senegal**	2014	64.5	56.6	59.5	64.8	71.7	70.0	60.0	69.8
**Swaziland**	2006	76.9	68.3	74.7	78.5	81.5	81.2	75.8	80.5
**Swaziland**	2010	80.1	74.7	76.4	82.1	82.3	84.1	78.9	83.7
**Uzbekistan**	1996	81.1	75.0	82.6	82.7	76.6	83.9	79.4	81.3
**Uzbekistan**	2006	83.2	79.7	83.9	82.8	84.1	85.6	83.6	81.9
**Vietnam**	1997	72.4	59.8	72.5	74.3	77.9	85.8	70.8	82.0
**Vietnam**	2002	79.0	67.0	76.9	82.9	85.8	90.4	77.4	86.8
**Vietnam**	2010	84.5	76.5	85.3	88.2	84.2	89.8	83.4	87.0
**Vietnam**	2013	85.5	74.8	89.2	87.9	89.4	88.8	84.6	87.8
**Yemen**	2006	47.3	31.4	36.5	45.9	56.0	70.7	40.1	64.5
**Yemen**	2013	51.6	33.8	43.4	51.5	60.9	70.5	45.5	66.5
**Zambia**	1996	66.3	57.9	62.0	64.2	69.9	80.0	60.9	74.4
**Zambia**	2001	66.6	58.2	60.0	63.8	73.4	80.4	62.0	75.9
**Zambia**	2007	69.3	67.2	62.5	63.8	76.0	81.1	65.4	78.0
**Zambia**	2013	75.6	68.5	70.5	74.5	82.5	85.6	71.2	83.6
**Upper-middle income countries**									
**Belize**	2006	68.9	59.9	68.2	71.2	74.3	73.2	66.1	71.7
**Belize**	2011	80.1	73.4	80.5	80.1	78.6	87.8	78.2	84.1
**Bosnia and Herzegovina**	2006	79.5	79.4	80.5	79.4	78.8	79.6	81.2	76.3
**Bosnia and Herzegovina**	2011	85.1	83.4	83.7	83.4	87.5	87.0	84.6	86.4

Table S3. Composite coverage index at national level and stratified by wealth quintiles and place of residence for each country and year of survey. (continued)

Country	Year	Composite coverage Index (%)							
		National	Wealth quintiles					Place of residence	
			Q1 (poorest)	Q2	Q3	Q4	Q5 (richest)	Rural	Urban
**Colombia**	1995	74.2	61.5	73.7	76.7	81.3	82.6	66.6	78.2
**Colombia**	2000	76.7	66.3	75.5	81.0	82.5	83.7	69.7	80.0
**Colombia**	2005	78.3	71.5	79.3	80.0	81.4	81.5	74.2	80.2
**Colombia**	2010	84.1	77.9	84.7	86.4	87.6	87.1	80.0	85.7
**Dominican Republic**	1996	70.2	62.8	68.6	72.6	73.9	75.6	66.3	72.8
**Dominican Republic**	1999	75.0	70.4	75.0	78.4	76.6	71.1	75.6	74.6
**Dominican Republic**	2002	77.8	74.6	76.3	79.2	80.6	77.7	77.1	78.2
**Dominican Republic**	2007	81.5	77.8	82.5	80.9	83.5	84.4	81.1	81.8
**Dominican Republic**	2013	82.1	79.7	85.3	80.2	82.7	81.8	83.7	81.5
**Gabon**	2000	61.1	45.2	57.6	65.4	67.9	69.4	47.9	65.4
**Gabon**	2012	68.8	59.1	67.1	68.7	76.3	73.0	59.8	70.3
**Jordan**	1997	77.9	74.7	77.0	79.0	78.9	79.5	75.2	78.5
**Jordan**	2002	80.3	78.3	80.0	80.8	80.4	81.3	77.4	81.0
**Jordan**	2007	82.8	79.4	82.6	85.0	82.5	84.6	82.4	82.9
**Jordan**	2012	84.1	82.5	83.6	85.0	83.9	85.7	85.3	83.9
**Kazakhstan**	1995	66.7	67.1	63.5	63.4	65.6	73.7	62.6	70.3
**Kazakhstan**	1999	76.7	73.4	76.1	79.0	77.9	77.1	76.1	77.0
**Kazakhstan**	2010	86.8	83.1	86.7	86.0	87.3	90.5	83.9	89.6
**Macedonia**	2005	70.5	66.3	75.4	64.5	68.7	77.0	68.8	71.9
**Macedonia**	2011	85.6	84.3	79.0	86.7	89.7	82.4	84.2	87.2
**Montenegro**	2005	79.1	77.4	80.5	82.0	80.3	78.8	82.3	77.7
**Montenegro**	2013	77.4	63.5	81.7	85.6	80.8	73.0	68.0	81.9

Table S3. Composite coverage index at national level and stratified by wealth quintiles and place of residence for each country and year of survey. (continued)

Country	Year	Composite coverage Index (%)							
		National	Wealth quintiles					Place of residence	
			Q1 (poorest)	Q2	Q3	Q4	Q5 (richest)	Rural	Urban
**Namibia**	2000	69.4	58.7	62.6	67.3	73.3	81.8	64.5	77.5
**Namibia**	2006	76.7	62.8	74.7	75.1	84.7	87.5	71.9	83.1
**Namibia**	2013	79.1	73.4	79.2	80.1	80.4	81.1	76.5	81.3
**Peru**	1996	67.8	50.9	65.2	74.4	79.7	81.6	56.1	75.4
**Peru**	2000	75.1	62.2	71.5	80.7	83.3	88.2	66.6	81.7
**Peru**	2004	81.0	69.6	78.0	83.4	88.3	89.9	73.9	86.2
**Peru**	2005	80.8	70.5	77.6	85.8	85.3	88.3	74.8	85.6
**Peru**	2006	81.7	70.4	77.7	85.7	89.3	92.1	75.5	86.3
**Peru**	2007	81.2	70.8	79.3	82.1	81.6	89.1	78.3	82.9
**Peru**	2008	81.8	72.2	78.5	81.9	85.7	87.7	77.1	84.7
**Peru**	2009	81.4	73.8	81.4	82.9	85.3	86.8	76.7	83.7
**Peru**	2010	82.2	74.8	81.9	84.8	86.0	87.1	76.8	85.1
**Peru**	2011	84.2	75.4	84.7	86.2	88.3	91.2	78.5	87.3
**Peru**	2012	83.9	77.3	82.7	86.9	88.0	87.7	79.6	86.1

Table S4. Time trends in the composite coverage index comparing (a) the poorest quintile (Q1) with the four richest quintiles (Q2-Q5), and (b) the two poorest (Q1-Q2) with the three wealthiest quintiles (Q3-Q4), by country income groups, 1994-2014. Slopes are expressed in percent points. Analyses weighted by national under-five populations.

Table S4.a. Comparison of Q1 with Q2-Q5.

Income group (World Bank)	Average national slope		Average slope in				Ratio Q1/ Q2-Q5 Slopes	Change in national slope due to Q1 (%)
			Q1		Q2-Q5			
	Slope	SE	Slope	SE	Slope	SE		
**Global**	0.85	0.06	0.95	0.12	0.81	0.06	1.17	5.0
**Low income**	1.06	0.13	1.12	0.14	1.01	0.14	1.10	4.3
**Lower middle income**	0.75	0.06	0.84	0.17	0.72	0.06	1.17	4.3
**Upper middle income**	0.79	0.09	1.19	0.14	0.67	0.09	1.78	15.7

Table S4.b. Comparison of Q1-Q2 with Q3-Q5.

Income group (World Bank)	Average national slope		Average slope in				Ratio Q1-Q2/ Q3-Q5 Slopes	Change in national slope due to Q1-Q2 (%)
			Q1-Q2		Q3-Q5			
	Slope	SE	Slope	SE	Slope	SE		
**Global**	0.85	0.06	1.00	0.09	0.73	0.07	1.37	15.3
**Low income**	1.06	0.13	1.13	0.14	0.99	0.14	1.14	6.8
**Lower middle income**	0.75	0.06	0.93	0.12	0.60	0.07	1.55	19.7
**Upper middle income**	0.79	0.09	1.09	0.12	0.53	0.09	2.06	32.4

Table S5. Trends in urban and rural areas in the composite coverage index, by country income groups, 1994-2014. Slopes are expressed in percent points. Analyses weighted by national under-five populations.

Income group (World Bank)	Average national slope		Average slope in				Ratio rural/urban slopes	Change in national slope due to rural areas (%)
			Rural		Urban			
	Slope	SE	Slope	SE	Slope	SE		
**Global**	0.85	0.06	0.87	0.10	0.51	0.07	1.71	40.6
**Low income**	1.06	0.13	1.12	0.12	0.64	0.12	1.75	39.7
**Lower middle income**	0.75	0.06	0.70	0.17	0.42	0.06	1.67	44.4
**Upper middle Income**	0.79	0.09	1.09	0.12	0.58	0.08	1.88	26.8

Table S6. Trends in the composite coverage index in the 40% poorest (Q1-Q2), the 60% richest (Q3-Q5), urban and rural areas, by country, 1994-2014. Slopes are expressed in percent points.

Country	Number of surveys	Average national slope		Average slope in				Average slope in			
				Q1-Q2		Q3-Q5		Urban		Rural	
		Slope	SE	Slope	SE	Slope	SE	Slope	SE	Slope	SE
**Armenia**	3	1.00	0.17	0.78	0.17	0.95	0.17	0.97	0.21	1.02	0.34
**Bangladesh**	4	1.38	0.09	1.47	0.09	1.23	0.09	1.04	0.15	1.39	0.10
**Belize**	2	2.22	0.36	2.59	0.36	1.74	0.36	2.47	0.49	2.43	0.50
**Benin**	4	0.18	0.10	0.29	0.10	0.01	0.10	0.04	0.14	0.16	0.12
**Bolivia**	4	1.42	0.08	2.03	0.08	0.97	0.08	1.01	0.09	2.05	0.11
**Bosnia and Herzegovina**	2	1.12	0.33	0.71	0.33	1.35	0.33	2.02	0.54	0.68	0.40
**Burkina Faso**	3	2.04	0.21	1.78	0.21	1.99	0.21	0.59	0.28	2.08	0.21
**CAR**	3	-0.18	0.10	-0.24	0.10	-0.21	0.10	-0.01	0.11	-0.29	0.11
**Cambodia**	4	2.42	0.08	2.79	0.08	2.10	0.08	1.93	0.15	2.50	0.09
**Cameroon**	4	0.34	0.16	0.35	0.16	0.32	0.16	-0.05	0.17	0.41	0.22
**Chad**	3	0.05	0.11	0.12	0.11	-0.03	0.11	-0.07	0.13	0.09	0.12
**Colombia**	4	0.68	0.04	0.95	0.04	0.49	0.04	0.49	0.04	0.93	0.08
**Comoros**	2	0.52	0.12	0.49	0.12	0.50	0.12	0.69	0.22	0.40	0.14
**Congo Brazzaville**	2	0.73	0.25	0.96	0.25	0.51	0.25	0.30	0.25	0.65	0.37
**Congo Democratic Republic**	3	0.52	0.24	0.58	0.24	0.49	0.24	0.52	0.30	0.69	0.32
**Cote dIvoire**	4	0.63	0.10	0.99	0.10	0.47	0.10	0.40	0.10	0.80	0.12
**Dominican Republic**	5	0.75	0.05	1.03	0.05	0.53	0.05	0.57	0.06	1.06	0.08
**Egypt**	5	0.77	0.03	1.16	0.03	0.50	0.03	0.40	0.04	1.07	0.04
**Ethiopia**	3	1.32	0.14	1.08	0.14	1.52	0.14	0.81	0.41	1.28	0.12
**Gabon**	2	0.64	0.10	0.98	0.10	0.43	0.10	0.41	0.09	0.99	0.19
**Ghana**	5	1.08	0.07	1.35	0.07	0.80	0.07	0.46	0.12	1.24	0.08

Table S6. Trends in the composite coverage index in the 40% poorest (Q1-Q2), the 60% richest (Q3-Q5), urban and rural areas, by country, 1994-2014. Slopes are expressed in percent points. (continued)

Country	Number of surveys	Average national slope		Average slope in				Average slope in			
				Q1-Q2		Q3-Q5		Urban		Rural	
		Slope	SE	Slope	SE	Slope	SE	Slope	SE	Slope	SE
**Guatemala**	2	1.88	0.73	1.51	0.73	1.75	0.73	1.23	1.10	2.03	0.91
**Guinea**	3	0.36	0.13	0.66	0.13	0.12	0.13	0.11	0.15	0.49	0.14
**Guyana**	2	1.73	0.61	1.70	0.61	1.68	0.61	1.10	1.09	1.86	0.72
**Haiti**	3	1.08	0.11	1.22	0.11	0.95	0.11	0.72	0.12	1.19	0.15
**Honduras**	2	1.24	0.10	1.77	0.10	0.80	0.10	0.67	0.14	1.61	0.12
**India**	2	0.92	0.09	1.19	0.09	0.74	0.09	0.50	0.16	0.94	0.12
**Indonesia**	4	0.67	0.05	0.89	0.05	0.48	0.05	0.29	0.08	0.77	0.07
**Jordan**	4	0.42	0.04	0.47	0.04	0.40	0.04	0.37	0.05	0.73	0.07
**Kazakhstan**	3	1.20	0.12	1.17	0.12	1.23	0.12	1.25	0.15	1.23	0.17
**Kenya**	3	0.08	0.12	0.07	0.12	-0.06	0.12	-0.20	0.24	0.13	0.13
**Kyrgyzstan**	4	0.03	0.10	0.02	0.10	0.03	0.10	-0.25	0.17	0.04	0.12
**Lesotho**	2	0.78	0.25	0.92	0.25	0.54	0.25	0.83	0.52	0.52	0.26
**Liberia**	2	1.73	0.31	2.92	0.31	0.89	0.31	0.00	0.32	2.16	0.39
**Macedonia**	2	2.51	0.38	1.92	0.38	2.88	0.38	2.54	0.45	2.57	0.49
**Madagascar**	3	1.52	0.16	1.58	0.16	1.63	0.16	1.52	0.22	1.73	0.19
**Malawi**	4	1.47	0.05	1.74	0.05	1.26	0.05	0.53	0.13	1.59	0.05
**Mali**	4	0.89	0.09	1.02	0.09	0.80	0.09	0.47	0.13	1.13	0.09
**Mauritania**	2	-0.16	0.30	0.37	0.30	-0.20	0.30	0.01	0.35	0.25	0.36
**Moldova**	2	0.40	0.19	0.68	0.19	0.19	0.19	0.02	0.29	0.58	0.29
**Mongolia**	2	1.05	0.19	1.01	0.19	0.79	0.19	1.02	0.26	0.88	0.34
**Montenegro**	2	-0.21	0.40	-0.73	0.40	-0.12	0.40	0.53	0.47	-1.78	0.64

Table S6. Trends in the composite coverage index in the 40% poorest (Q1-Q2), the 60% richest (Q3-Q5), urban and rural areas, by country, 1994-2014. Slopes are expressed in percent points. (continued)

Country	Number of surveys	Average national slope		Average slope in				Average slope in			
				Q1-Q2		Q3-Q5		Urban		Rural	
		Slope	SE	Slope	SE	Slope	SE	Slope	SE	Slope	SE
**Mozambique**	3	0.29	0.15	0.34	0.15	0.13	0.15	-0.02	0.16	0.40	0.18
**Namibia**	3	0.68	0.09	1.17	0.09	0.42	0.09	0.21	0.13	0.88	0.11
**Nepal**	5	1.75	0.07	1.83	0.07	1.70	0.07	1.25	0.14	1.75	0.07
**Nicaragua**	2	1.99	0.33	2.93	0.33	1.25	0.33	1.28	0.31	3.07	0.53
**Niger**	3	1.66	0.12	1.79	0.12	1.48	0.12	0.63	0.14	1.87	0.12
**Nigeria**	5	0.75	0.17	0.27	0.17	1.18	0.17	2.98	0.22	-0.48	0.19
**Pakistan**	2	1.04	0.21	1.29	0.21	1.00	0.21	1.01	0.30	1.11	0.26
**Peru**	11	0.93	0.04	1.35	0.04	0.48	0.04	0.61	0.04	1.35	0.06
**Philippines**	4	0.44	0.06	0.72	0.06	0.18	0.06	0.16	0.08	0.70	0.08
**Rwanda**	3	2.36	0.08	2.50	0.08	2.19	0.08	1.70	0.21	2.45	0.08
**Senegal**	4	0.97	0.15	1.22	0.15	0.76	0.15	0.67	0.17	1.10	0.16
**Sierra Leone**	3	2.77	0.26	3.38	0.26	2.40	0.26	2.16	0.36	3.07	0.31
**Swaziland**	2	0.79	0.25	0.98	0.25	0.59	0.25	0.80	0.48	0.80	0.29
**Tajikistan**	2	0.84	0.28	1.31	0.28	0.66	0.28	0.59	0.38	0.93	0.36
**Tanzania**	4	0.82	0.10	0.95	0.10	0.77	0.10	0.59	0.14	0.85	0.11
**Togo**	4	0.62	0.10	0.62	0.10	0.53	0.10	0.31	0.12	0.49	0.12
**Uganda**	4	0.89	0.06	0.79	0.06	0.91	0.06	0.48	0.12	0.92	0.07
**Uzbekistan**	2	0.21	0.16	0.32	0.16	0.32	0.16	0.05	0.20	0.42	0.34
**Vietnam**	4	0.79	0.10	1.02	0.10	0.52	0.10	0.29	0.15	0.85	0.11
**Yemen**	2	0.61	0.23	0.66	0.23	0.58	0.23	0.28	0.28	0.77	0.23
**Zambia**	4	0.58	0.06	0.66	0.06	0.54	0.06	0.54	0.07	0.64	0.07
**Zimbabwe**	5	0.26	0.04	0.35	0.04	0.20	0.04	0.09	0.07	0.31	0.05

Table S7. Pearson correlation matrix for CCI and the eight separate indicators included in this index, in the most recent survey in the 64 countries under study.

	CCI	FPS	ANC	SBA	BCG	MSL	DPT3	CAREP	ORT
**CCI**	1.00								
**FPS**	0.79***	1.00							
**ANC**	0.72***	0.21	1.00						
**SBA**	0.82***	0.43**	0.73***	1.00					
**BCG**	0.82***	0.37*	0.71***	0.66***	1.00				
**MSL**	0.86***	0.47***	0.65***	0.69***	0.90***	1.00			
**DPT3**	0.86***	0.48***	0.65***	0.65***	0.91***	0.93***	1.00		
**CAREP**	0.78***	0.60***	0.56***	0.57***	0.57***	0.57***	0.57***	1.00	
**ORT**	0.52***	0.18	0.37**	0.37**	0.42***	0.41***	0.44***	0.24	1.00

NOTE: FPS: Family planning satisfied; ANC: antenatal care; SBA: skilled attendant at birth; BCG vaccine; MSL: Measles vaccine; DPT3 vaccine; CAREP: careseeking for symptoms of pneumonia; ORT: oral rehydration therapy

*** p<0.001

** 0.001<p<0.01

* 0.01<p<0.5

Figures

Figure S1. Regression lines for annual changes in CCI in the poores[illegible] Q2) and richest 60% (Q3-Q5) of the population, by country inco[illegible] showing confidence intervals.

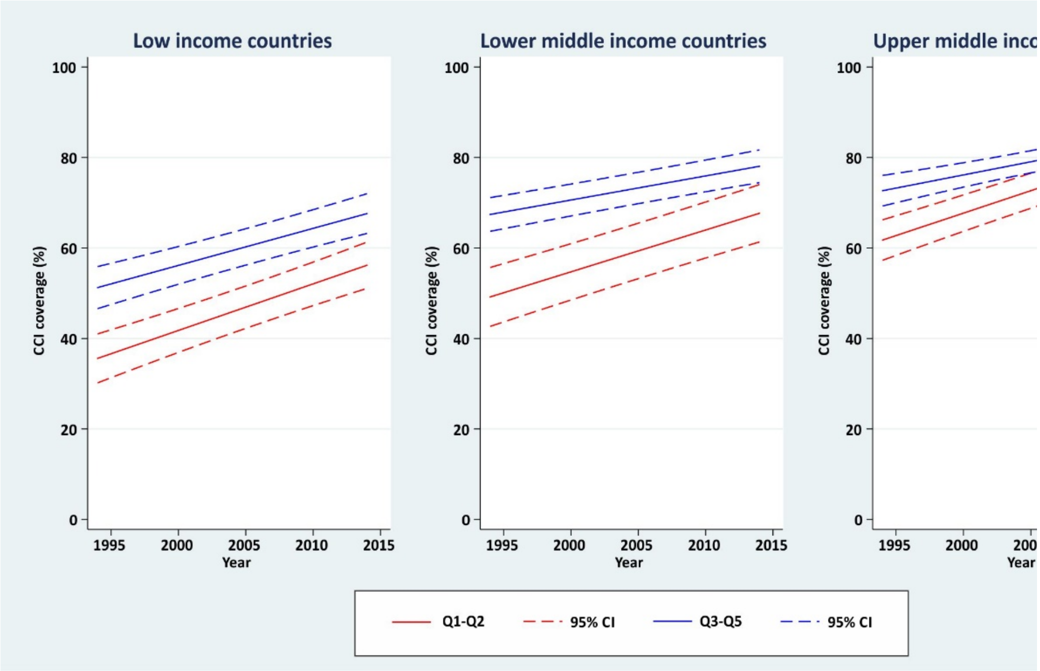

Figure S2. Regression lines for annual changes in CCI in rural and urban areas, by country income groups, showing confidence intervals.

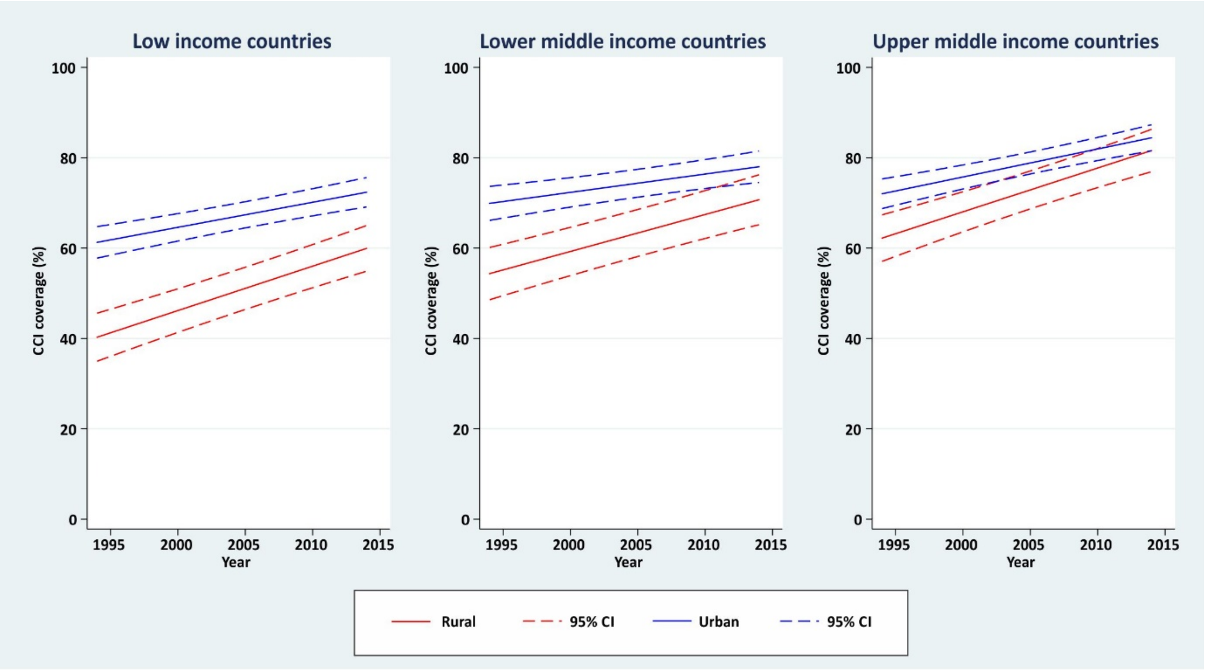

Figure S3. Regression lines for annual changes in CCI according to wealth quintiles, by country income groupings (low, lower middle and upper middle income). Analyses weighted by national under-five populations. Comparison in the poorest 40% (Q1-Q2) and richest 60% (Q3-Q5).

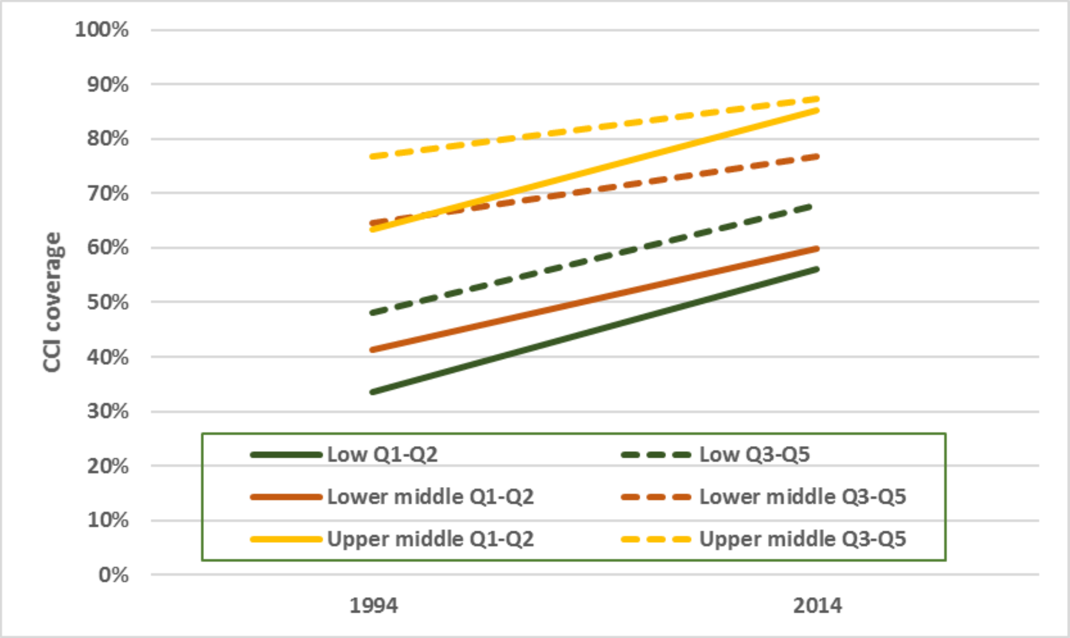

Figure S4. Regression lines for annual changes in CCI according in rural and urban areas, by country income groupings. Analyses weighted by national under-five populations.

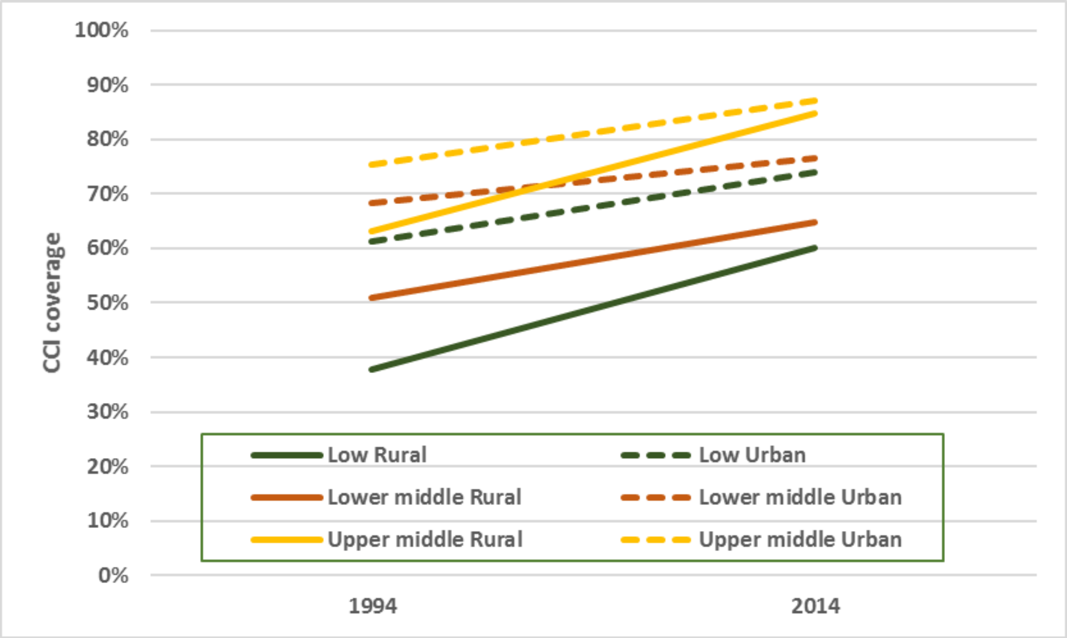

Figure S5. Time trends in CCI and CIX for all countries studied, 1994-2014. Analyses weighted by national under-five populations.

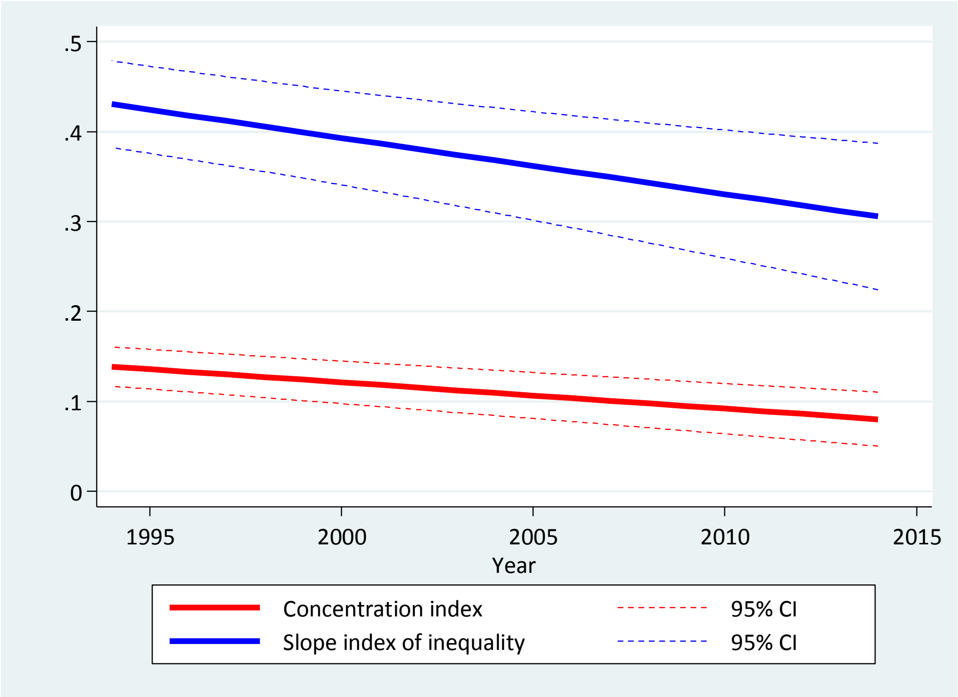

Supplement: Supplementary appendix [file mmc1.pdf]
